# Access to sexual and reproductive health commodities in East and Southern Africa: a cross-country comparison of availability, affordability and stock-outs in Kenya, Tanzania, Uganda and Zambia

**DOI:** 10.1186/s12889-020-09155-w

**Published:** 2020-07-03

**Authors:** Gaby I. Ooms, Denis Kibira, Tim Reed, Hendrika A. van den Ham, Aukje K. Mantel-Teeuwisse, Gemma Buckland-Merrett

**Affiliations:** 1 Health Action International, Overtoom 60-2, 1054 HK Amsterdam, The Netherlands; 2 WHO Collaborating Centre for Pharmaceutical Policy and Regulation, Division of Pharmacoepidemiology and Clinical Pharmacology, Utrecht Institute for Pharmaceutical Sciences (UIPS), Utrecht University, Utrecht, The Netherlands; 3 Coalition for Health Promotion and Social Development (HEPS-Uganda), Kampala, Uganda; 4 Wellcome Trust, London, UK

**Keywords:** Sexual and reproductive health, Family planning, Maternal health, Newborn and child health, Sexually transmitted infections, Availability, Affordability, Accessibility, Health systems

## Abstract

**Background:**

Access to sexual and reproductive health services continues to be a public health concern in Kenya, Tanzania, Uganda and Zambia: use of modern contraceptives is low, and unmet family planning needs and maternal mortality remain high. This study is an assessment of the availability, affordability and stock-outs of essential sexual and reproductive health commodities (SRHC) in these countries to inform interventions to improve access.

**Methods:**

The study consisted of an adaptation of the World Health Organization/Health Action International methodology, *Measuring Medicine Prices, Availability, Affordability and Price Components*. Price, availability and stock-out data was collected in July 2019 for over fifty lowest-priced SRHC from public, private and private not-for-profit health facilities in Kenya (*n* = 221), Tanzania (*n* = 373), Uganda (*n* = 146) and Zambia (*n* = 245). Affordability was calculated using the wage of a lowest-paid government worker. Accessibility was illustrated by combining the availability (≥ 80%) and affordability (less than 1 day’s wage) measures.

**Results:**

Overall availability of SRHC was low at less than 50% in all sectors, areas and countries, with highest mean availability found in Kenyan public facilities (46.6%). Stock-outs were common; the average number of stock-out days per month ranged from 3 days in Kenya’s private and private not-for-profit sectors, to 12 days in Zambia’s public sector. In the public sectors of Kenya, Uganda and Zambia, as well as in Zambia’s private not-for-profit sector, all SRHC were free for the patient. In the other sectors unaffordability ranged from 2 to 9 SRHC being unaffordable, with magnesium sulphate being especially unaffordable in the countries. Accessibility was low across the countries, with Kenya’s and Zambia’s public sectors having six SRHC that met the accessibility threshold, while the private sector of Uganda had only one SRHC meeting the threshold.

**Conclusions:**

Accessibility of SRHC remains a challenge. Low availability of SRHC in the public sector is compounded by regular stock-outs, forcing patients to seek care in other sectors where there are availability and affordability challenges. Health system strengthening is needed to ensure access, and these findings should be used by national governments to identify the gaps and shortcomings in their supply chains.

## Background

Worldwide, more than 800 women a day die due to complications related to pregnancy and childbirth, and annually an estimated 5.3 million children do not reach the age of five, with half of these deaths occurring in sub-Saharan Africa [1, 2]. In addition to the threat of death, 210 million women a year experience serious pregnancy-related injuries and disabilities, which often lead to long-term morbidity [3]. Research has estimated that the lives of four million women, newborns and children in sub-Saharan Africa could be saved if coverage of interventions such as emergency obstetric care, breastfeeding counselling, and treatment for infections such as diarrhoea and pneumonia increased to 90% of families [4]. Contraceptive prevalence rates remain low in many developing countries among both men and women, with over 214 million women experiencing unmet family planning needs, and the limited demand and uptake of reproductive health services and education around reproductive health issues pose significant challenges [5–8]. In addition, it is estimated that in 2020 there will be an annual shortfall of $233 million needed to pay for contraceptive supplies [6]. In 2016 alone, there were also an estimated 376 million new cases of one of the four most common curable sexually transmitted infections (STIs) (chlamydia, gonorrhoea, syphilis and trichomoniasis), with syphilis responsible for more than 200,000 stillborn and newborn deaths [9]. Access to essential commodities and services for sexual and reproductive health (SRH) can prevent a significant proportion of these deaths and disabilities. However, access remains a problem for almost 2 billion people [10].

Reflecting global trends, access to SRH services continues to be a public health concern in Kenya, Tanzania, Uganda and Zambia. Ranging from 224 to 510 maternal deaths per 100,000 live births, the maternal mortality in these countries remains high, especially when comparing it to the maternal mortality rate in developed countries (12 per 100,000 live births) [11, 12]. The use of modern contraceptives is low, especially in Uganda and Tanzania, where only 27.5 and 33.5% of married women, respectively, used modern contraceptive methods. In Kenya, Tanzania and Zambia, about 20% of married women aged 15–49 had unmet family planning needs, while 30% of married women in Uganda were experiencing this problem [7]. In Zambia, 81.9% of unmarried, sexually active adolescent girls aged 15–19 were not using contraception [13]. The other three countries also have high percentages of unmarried, sexually active adolescent girls not using contraception (59.3 to 68.8%) [14–16]. Not surprisingly, overall unmet needs for contraceptives among this population was high; across the four countries it ranged from 38.6 to 66.9% [14, 17]. Consequences of unmet family planning needs can be serious, especially amongst adolescents: it can lead to unwanted pregnancies, unsafe abortions, and increased risks for morbidity and mortality [18]. Further, teenage pregnancies can lead to school dropout, which diminishes the chances of girls finding employment opportunities later in life, continuing the poverty cycle [18]. Significant changes are thus needed to reach the Sustainable Development Goals’ targets of a global maternal mortality ratio of less than 70 per 100,000 live births and universal access to sexual and reproductive healthcare services [19].

Despite the clear need for access to sexual and reproductive health commodities (SRHC) in Kenya, Tanzania, Uganda and Zambia, access has not been fully achieved and unavailability, unaffordability, regulatory provisions and supply chain issues persist [20]. Previous research in these countries has focused on identifying the barriers to access on both the supply and demand side [21–26], but detailed research on availability and affordability of these medicines at the health system level is lacking. In Uganda research on availability of medicines for SRH has been conducted previously, showing that access remains suboptimal [27, 28]. However, this research did not cover an extensive list of SRHC, nor included medical devices essential in offering quality SRH services. The research presented here is an assessment of the availability, affordability and stock-outs of over fifty essential SRHC, including medicines and medical devices, in Kenya, Tanzania, Uganda and Zambia to identify current accessibility of SRHC and to inform interventions to improve access.

## Methods

### Study design

The study was designed as a cross-sectional survey. Data collection comprised a health facility survey in which the availability, price, and stock-outs of SRHC were measured.

Ethical approval was granted by the Amref Ethics and Scientific Review Committee in Kenya, the National Institute for Medical Research in Tanzania, Makerere University School of Health Sciences in Uganda, and the National Health Research Authority in Zambia. Letters of introduction to health facilities were provided by County Directors of Health in Kenya, and Ministries of Health in Tanzania, Uganda and Zambia.

### Study setting and participants

This survey was conducted in ten counties in Kenya, twelve counties in Tanzania, six regions in Uganda, and ten provinces in Zambia. The provinces selected included each country’s main urban region and five or more other regions, using a random sampling strategy. Each survey area within a province covered a population of 100,000 to 250,000. Health facilities were identified for inclusion, using a stratification method, as public-, private-, and private not-for-profit (PNFP) facilities. Within each stratum, four health facilities were randomly sampled from rural and urban areas. In this study urban areas were defined per country according to the definition held by the corresponding National Bureaus of Statistics: an urban area was defined in Kenya and Uganda as an area with a population of 2000 or higher, in Zambia with a population of 5000 or higher, and in Tanzania with a population of 10,000 or higher [29]. In each case, one of the selected urban areas included the main public provincial health facility. The inclusion criteria for the other health facilities were that facilities had to be within 3 h travel from the main public provincial health facility, and all selected health facilities had to provide SRH services.

### Data collection tool

A data collection tool, adapted from the standardised World health Organization (WHO)/Health Action International (HAI) Medicine Prices Monitoring Tool and validated in many countries, was used for collecting data [30–34]. The ‘basket’ of commodities assessed was developed by combining the WHO’s Essential Medicines for Reproductive Health, the Interagency List of Essential Medicines for Reproductive Health, the Interagency List of Medical Devices for Essential Interventions for Reproductive, Maternal, Newborn and Child Health, and the United Nations Commission on Life Saving Commodities for Women and Children: Commissioner’s Report [35–38]. In combination with in-country expertise via a specialist advisory group and after piloting the methodology, after which slight alterations were made to the commodity basket, the commodities list presented was believed to be a selection of the most essential SRHC within the study region. Commodity strengths and dosage forms were based on the national essential medicine lists (NEMLs) [39–43]. Commodities cover family planning, maternal and child health, and STI management, and when listed with multiple dosage forms or strengths, all the formulations were included in the survey (see Additional file 1 for a complete overview of surveyed commodities). Previous cycles of the research took place in 2017 and 2018 in Kenya, Tanzania, Uganda and Zambia.

### Data collection

Data collection took place in July 2019 using a mobile data collection application. In each country, local data collectors were trained by the authors (GIO and DK) on how to use the data collection tool during a two-day workshop organised by Health Action International, which included a field test. During the workshop the data collectors were provided with one tablet each and taught how to use the mobile application through a step-by-step walkthrough. During the field test they practiced the use of the mobile application.

Data collectors worked in pairs, supervised in each country by a survey manager. Data on availability, patient prices, brand information and stock-out days was only collected when commodities were visibly present. Product name, name of manufacturer, actual pack size and pack price were recorded for the lowest price for each commodity available. Stock-outs were only recorded if a stock card was available and seen. Stock-outs were noted for the 6 months prior to the day of data collection.

### Data analysis

After completion of data collection, data was uploaded to the server and downloaded into an excel spreadsheet. Data entries were double-checked for accuracy by the survey managers and researchers. If data was incompletely or incorrectly entered, such as if a wrong product or pack size was noted, or a wrong unit price was calculated, the data was rectified after verification with the data collectors or or an ‘X’ was noted to denote only the availability of the commodity when pricing information could not be verified. Thereafter, analysis was completed in a previously developed Excel analysis tool using descriptive statistics.

The availability of a commodity was calculated as the mean of the sampled facilities where the medicine was found at the time of the survey, expressed as a percentage. Mean availability of SRHC per sector and country was calculated in a two-step manner: firstly, the mean availability per commodity across the sampled facilities was calculated, after which the mean of these mean availabilities was calculated. For each commodity, availability was only measured when the level of care at which a commodity should be available corresponded with the surveyed facility. For example, calcium gluconate should be available at hospital levels and up in Kenya, Tanzania and Zambia, and from health centre III level in Uganda. In the PNFP sector, availability of family planning commodities was only calculated if family planning services were provided by the facility. Availability was calculated per commodity, as well as in groups for similar use (the birth control pill, injectable contraceptive and implant) or for different formulations of the same medicine (i.e. for magnesium sulphate, amoxicillin, clotrimazole, ferrous salt, folic acid, zinc and ORS sachets). When availability was calculated for a grouping of commodities, it was an aggregate of the availability and calculated as the mean percentage of sampled facilities where either of the formulations or commodities with similar medicinal use were available. Availability of 80% or higher was considered acceptable as per WHO guidelines [44]. Two-sample F-tests for variance were computed to test for normal distribution and independence, after which two-sample t-tests were calculated to test whether significant differences existed between means, using a significance cut-off value of 0.05.

Stock-outs were calculated longitudinally as the mean percentage of facilities that reported a stock-out of a commodity any time in the 6 months prior to the day of data collection. Stock-out days were also calculated longitudinally over a six-month period and were calculated as the average number of days a commodity was stocked out per month. Stock information was surveyed only for medicines, not for medical devices.

Affordability was calculated using the median price of a commodity, and the number of days a lowest-paid government worker (LPGW) needs to work in order to pay for a standard treatment regimen for a commodity. The daily wage of an LPGW was 449.40 Kenyan Shillings (Kenya), 3077.15 Tanzanian Shillings (Tanzania), 6169.65 Ugandan Shillings (Uganda), and 33.12 Kwacha (Zambia) [45–48]. According to the WHO/HAI methodology, treatment was considered unaffordable if it cost more than a day’s wage for an LPGW [30]. Affordability was calculated only for medicines, not for medical devices.

Accessibility was illustrated combining the availability and affordability measures. This resulted in a categorical variable, in which accessibility was achieved when a commodity had an 80% or higher availability, and when a treatment regimen cost less than a day’s wage of an LPGW.

## Results

Across the public, private and PNFP sectors, 221, 373, 146 and 245 facilities were surveyed in Kenya, Tanzania, Uganda and Zambia, respectively. Stock information was collected from 221 facilities in Kenya, 212 facilities in Tanzania, 105 facilities in Uganda, and 182 facilities in Zambia. An overview of the distribution of the facilities is provided in Table 1.

**Table 1:** Distribution of surveyed facilities with availability, price and stock data, by country, sector and area

	Public	Private	PNFP	Total
Kenya				
Availability and price data				
Urban	33	63	24	120
Rural	46	25	30	101
Total	79	88	54	221
Stock data				
Urban	33	63	24	120
Rural	45	25	30	100
Total	78	88	54	220
Tanzania				
Availability and price data				
Urban	131	55	35	221
Rural	132	5	15	152
Total	263	60	50	373
Stock data				
Urban	100	25	21	146
Rural	56	1	9	66
Total	156	26	30	212
Uganda				
Availability and price data				
Urban	22	33	23	78
Rural	33	15	20	68
Total	55	48	43	146
Stock data				
Urban	21	16	21	58
Rural	29	0	18	47
Total	50	16	39	105
Zambia				
Availability and price data				
Urban	59	58	4	121
Rural	77	9	38	124
Total	136	67	42	245
Stock data				
Urban	48	30	4	82
Rural	57	5	38	100
Total	105	35	42	182

*PNFP* Private not-for-profit

### Availability of SRHC

#### Across countries

The research surveyed 55 commodities in Kenya, 56 in Tanzania and Zambia, and 59 in Uganda. Aggregation led to 43 surveyed SRHC in all countries. Mean availability of SHRC in general on the day of data collection was lower than 50% in all sectors. Highest mean availability was found in Kenya for all sectors, with the highest overall mean availability found in Kenya’s public sector (46.6%). Mean availability in Tanzania’s (37.9%), Uganda’s (37.9%) and Zambia’s (38.6%) public sectors was comparable to each other. Zambia’s private sector had the lowest mean availability across the countries and sectors (28.3%). Comparing the countries to each other showed that mean availability of SHRC in the PNFP sector was significantly higher in Kenya (45.7%, *n* = 55) than in Tanzania (33.5%, *n* = 56) (*p* = 0.01). No significant differences in mean availability were found across the countries for any other sectors.

#### Country level

**Fig. 1:**
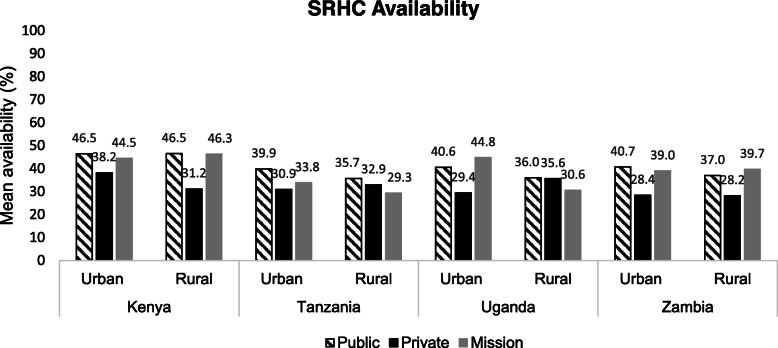
Mean availability of SRHC across sectors and areas, by country

In none of the countries did the mean availability of SHRC differ significantly between sectors. In Uganda mean availability within the PNFP sector differed significantly when comparing urban and rural facilities: mean availability of SRHC in urban PNFP facilities (44.8%, *n* = 59) was significantly higher than in rural PNFP facilities (30.6%, *n* = 59) (*p* = 0.009) (Fig. 1). There were no significant differences in mean availability when comparing urban and rural areas within a sector in the other countries.

In all countries, the public sector had the most commodities with an 80% availability or more. Kenya’s public sector had 10 SRHC with an 80% or higher availability, followed by Zambia (8 SRHC), and Uganda and Tanzania (both 6 SRHC) (see Table 1). In all countries, the private sector had the most commodities available at 50% or less of facilities: 25 of 43 SRHC in Kenya, 27 of 43 SRHC in Uganda, 30 of 43 SRHC in Tanzania, and 33 of 43 SRHC in Zambia.

#### Family planning

In the countries, male condoms were most likely to be available in more than 80% of the facilities across the different sectors (see Table 2). Only in Kenya’s and Tanzania’s PNFP sector, and Tanzania’s and Uganda’s private sector was the availability below 80%. Female condoms were available at 60% or less of the facilities across the countries. Kenya’s public sector had the most family planning commodities available at more than 80% of facilities, this included the combination measures of oral contraceptive, injectable contraceptive and the implant. Levonorgestrel 750 mcg, an emergency contraceptive, had a low availability across the countries, with Tanzania only providing it in 8% of public facilities, and in none of the private or PNFP facilities.

**Table 2:** Mean availability of individual SRHC across sectors, by country

Commodities	Mean Availability (%)											
	Kenya			Tanzania			Uganda			Zambia		
	Public	Private	PNFP	Public	Private	PNFP	Public	Private	PNFP	Public	Private	PNFP
Family Planning												
Oral contraceptive^a^	91	80	72	69	47	40	75	36	43	91	67	83
Levonorgestrel 750 mcg	47	71	32	8	0	0	35	30	24	41	61	41
Injectable contraceptive^b^	92	32	66	82	59	53	62	51	67	72	30	79
Male condoms	84	85	79	82	52	47	93	72	81	89	89	86
Female condoms	56	13	39	33	22	27	18	17	33	60	5	48
Intrauterine contraceptive device^n^	82	29	48	62	65	53	72	39	56	51	2	41
Implant^c,n^	87	30	79	84	78	73	89	64	67	71	0	62
Diaphragm	0	0	4	0	0	0	0	0	0	0	0	0
Maternal Health												
Oxytocin injection^n,p^	87	41	67	75	42	56	80	50	64	94	50	78
Misoprostol	33	34	35	49	35	22	82	48	56	11	27	26
Methyldopa^k,m,n^	77	77	83	76	73	73	24	14	36	12	51	17
Magnesium sulphate^d,n,p^	59	10	44	70	18	48	69	10	42	13	0	12
Calcium gluconate^k,m,n,q^	71	29	71	44	0	44	43	8	27	67	0	0
Ferrous salt	57	50	43	5	3	4	4	27	37	75	55	71
Folic acid	55	77	79	63	50	48	64	71	63	80	82	79
Ferrous Salt: Folic Acid Tablet^g^	62	8	48	20	5	14	27	10	33	1	7	2
Antibiotics and Antifungals												
Metronidazole	68	80	87	61	92	86	75	94	84	88	87	93
Clotrimazole^e^	70	74	83	39	47	38	67	63	67	24	45	17
Gentamicin^n^	58	39	61	43	45	40	30	67	67	48	33	50
Procaine benzylpenicillin	NS	NS	NS	26	33	36	33	48	44	2	34	7
Benzathine benzylpenicillin^n^	39	16	30	80	60	70	70	72	67	60	69	74
Benzylpenicillin	46	34	50	NS	NS	NS	NS	NS	NS	82	34	86
Amoxicillin^f^	62	38	43	74	30	42	40	40	49	77	88	81
Newborn- and Child Health												
Dexamethasone^k,o,q^	81	82	63	10	27	32	76	50	46	67	50	0
Zinc ORS co-pack	68	28	41	29	3	4	44	6	26	13	5	17
Zinc^h^	86	82	93	36	75	74	44	70	67	53	46	60
ORS sachets^i^	77	73	83	75	72	82	53	70	63	32	57	48
Chlorhexidine 4%	38	9	30	7	3	6	26	6	19	6	3	10
SRH medical devices												
Vasectomy kit^k,l,n^	19	6	8	3	5	0	17	8	15	7	4	14
Tubal ligation kit^k,l,n^	23	35	13	13	9	5	17	11	24	10	1	10
Manual vacuum aspiration kit^j,n^	66	59	56	38	38	26	48	36	48	54	38	64
Speculum^j,o^	86	78	84	84	67	66	88	75	92	84	38	94
Cervical dilator^j,n^	34	41	49	12	17	14	46	31	58	28	25	25
Incubator	68	47	79	11	13	16	16	4	14	27	13	53
Monitor^k,n^	39	53	63	11	15	16	22	11	33	14	13	17
Ultrasound scan^k,o^	45	53	63	13	45	36	52	63	92	19	50	22
Ventilator^k,n^	32	41	42	5	5	6	9	8	3	9	25	17
Foetal scope^j,n^	82	78	88	97	80	92	94	92	91	86	63	78
Resuscitator (adult size)^j,n^	45	33	49	31	22	18	33	44	45	28	25	39
Resuscitator (infant size)^j,n^	63	44	53	63	38	46	65	56	70	50	38	72
Bag and mask (size 0)^j,o^	58	52	77	59	37	46	68	25	62	52	38	75
Suction device^j^	68	67	79	71	52	62	65	54	81	75	50	81
Training mannequin (infant)^j,n^	20	15	23	36	22	32	26	6	27	26	0	33

*NS* not surveyed

^a^‘Oral contraceptive’ combines availability of ethinylestradiol + levonorgestrel (multiple formulations) and/or ethinylestradiol + norethisterone (multiple formulations) and/or ethinylestradiol + desorgestrel (multiple formulations) and/or levonorgestrel 30mcg at the facility

^b^‘Injectable contraceptive’ combines availability of medroxyprogesterone acetate (150 mg in 1 ml vial or 104 mg in 1 ml vial) and/or norethisterone enanthate 200 mg/ml in 1 ml vial and/or estradiol cypionate + medroxyprogesterone acetate (5 mg + 25 mg) at the facility

^c^‘Implant’ combines availability of levonorgestrel implant and/or etonogestrel implant at the facility

^d^‘Magnesium sulphate’ combines availability of magnesium sulphate 500 mg in 1 ml and/or magnesium sulphate 500 mg in 2 ml and/or magnesium sulphate 500 mg in 10 ml at the facility

^e^‘Clotrimazole’ combines availability of clotrimazole cream (1%, 15 g tube) and/or clotrimazole pessary (100 mg, 200 mg or 500 mg)

^f^‘Amoxicillin’ combines availability of amoxicillin 125 mg and/or amoxicillin 250 mg at the facility

^g^‘Ferrous salt: folic acid tablets’ combines availability of the ferrous salt: folic acid (60 mg + 400mcg) and/or ferrous salt: folic acid (150 mg + 500mcg) and/or ferrous salt: folic acid (200 mg + 500mcg) at the facility

^h^‘Zinc’ combines availability of zinc 10 mg in 5 ml syrup and/or zinc 20 mg and/or zinc ORS co-pack at the facility

^i^‘ORS sachets’ combines availability of ORS sachets of 200 ml and/or 500 ml and/or 1 L and/or zinc ORS co-pack at the facility

^j^Available from health centre and up in Kenya

^k^Available from primary/county hospital and up in Kenya

^l^Available from health centre and up in Tanzania

^m^Available from council hospital and up in Tanzania

^n^Available from health centre III and up in Uganda

^o^Available from health centre IV and up in Uganda

^p^Available from general hospital and up in Zambia

^q^Available from central hospital and up in Zambia

#### Maternal health

Maternal health commodities were on average less available than family planning commodities. Oxytocin only had an 80% or higher availability in the public sectors of Kenya, Uganda and Zambia. Misoprostol had a low availability across the countries; only in Uganda’s public sector was availability above 80%. Zambia had lowest availability across the sectors, ranging from 11 to 27%. Methyldopa had a relative high availability in all sectors in Kenya and Tanzania, while in Uganda and Zambia it was much lower. Magnesium sulphate had a low availability across the countries, especially in Zambia and the countries’ private sector.

#### Antibiotics and antifungals

In all countries, metronidazole had the highest availability in facilities. In Zambia, all sectors had an 80% or higher availability, while an 80% or higher availability was also found for the private and PNFP sectors in the other countries. Availability of clotrimazole, either the pessary or cream formulation, was considerably low in Tanzania and Zambia (less than 50% across the sectors), and only the PNFP sector in Kenya had either formulation available at more than 80% of facilities. Similarly, amoxicillin (125 mg or 250 mg), had a low availability in the countries; only in Zambia’s private and PNFP sector did the availability go above 80%. The benzylpenicillins had a suboptimal availability in most of the countries’ sectors.

#### Newborn and child health

Kenya had the best availability of newborn and child health commodities. Zinc had an 80% or higher availability across the sectors, while dexamethasone and ORS sachets also had a high availability. Overall, ORS sachets had the highest availability across the countries, with the exception of Zambia where availability was below 50% in the public and PNFP sectors. Chlorhexidine 4% had a low availability across all countries, with highest availability in Kenya’s public sector (38%).

#### SRH medical devices

Availability of SRH medical devices was generally low, with Kenya doing slightly better than the other countries. In all countries, availability of the vasectomy kit, tubal ligation kit, ventilator, resuscitator and infant-size training mannequin was below 50%. In Zambia’s private sector, all commodities, with the exception of the foetal scope, were available at less than 50% of facilities. Availability of the foetal scope was also high in the other countries.

### Stock-outs

Stock-out data was collected for 41 SRHC in Zambia, 42 SRHC in Kenya and Tanzania, and 45 SRHC in Uganda. Zambia had the highest percentage of SRHC stock-outs across the sectors. In the public sector, an average of 46.9% of facilities reported stock-outs, compared with 35.6% in Uganda, 25.1% in Tanzania and 23.2% in Kenya (see Table 3). In the private sector stock-outs occurred less often than in the public sector in Kenya and Uganda, while in Tanzania stock-outs occurred more often. Zambia’s stock-outs in the private sector were similar to the public sector. Stock-outs in the PNFP sector were much higher in Zambia than in the other three countries.

**Table 3:** Percentage of facilities reporting stock-outs in the last 6 months, and number of stock-out days per month

	Stock-outs	
	Facilities reporting stock-outs (%)	Average number of stock-out days/month
Kenya		
Public	23.2	6
Private	17.4	3
PNFP	12.0	3
Tanzania		
Public	25.1	8
Private	31.4	5
PNFP	14.5	9
Uganda		
Public	35.6	7
Private	16.6	4
PNFP	15.9	6
Zambia		
Public	46.9	12
Private	45.7	9
PNFP	41.7	10

*PNFP* Private not-for-profit

The average duration of stock-outs was also highest in Zambia, where stock-outs lasted 9 to 12 days per month across sectors. Stock-out duration in the public and private sectors of Kenya, Tanzania and Uganda were similar, ranging from 6 to 8 days per month in the public sector, and 3 to 5 days in the private sector. Tanzania’s PNFP sector stock-outs were comparable to Zambia’s, while in Uganda and Kenya they were lower.

### Affordability

Pricing information was missing for 0.6% (17/2946) of SRHC in Uganda, 1.1% (48/4469) of SRHC in Zambia, 2.5% (110/4316) of SRHC in Kenya and 6.5% (473/7289) of SRHC in Tanzania. In Kenya, Uganda and Zambia’s public sector all commodities were affordable to the patient because commodities were provided for free (see Table 4). Zambia’s PNFP sector also provided all SRHC for free to the patient. In Tanzania’s public sector, two SRHC cost more than a day’s wage for an LPGW: 2.27 days for a treatment of procaine benzylpenicillin, and 1.30 days for a treatment of gentamicin.

**Table 4:** Affordability of SRHC for an LPGW, per country and sector

		**Affordability (Days of Wages)**											
**Commodities**	**Treatment regimens**	**Kenya**			**Tanzania**			**Uganda**			**Zambia**		
		**Public**	**Private**	**PNFP**	**Public**	**Private**	**PNFP**	**Public**	**Private**	**PNFP**	**Public**	**Private**	**PNFP**
Ethinylestradiol + levonorgestrel	1 strip	0	0.13	0	0	0	0	0	0.16	0	0	0.30	0
Ethinylestradiol + norethisterone	1 strip	0	0.09	NA	0	0.19	NA	0	NA	NA	0	NA	NA
Ethinylestradiol + desorgestrel	1 strip	NA	NA	NA	0	0	NA	NA	NA	NA	NA	NA	NA
Levonorgestrel 30mcg	1 tablet	0	0.09	0.04	0	0	0	0	NA	NA	0	0.30	0
Levonorgestrel 750mcg	1 tablet	0	0.22	0.07	0	NA	NA	0	1.13	0.32	0	0.80	0
Medroxyprogesterone acetate 150 ml	1 vial	0	0.22	0.09	0	0	0	0	0.81	0	0	0.60	0
Medroxyprogesterone acetate 104 ml	1 vial	0	0.22	0.06	NA	NA	NA	0	0.32	0.08	NA	NA	NA
Norethisterone enanthate	1 vial	NA	NA	NA	NA	NA	NA	NA	NA	NA	0	0.63	0
Male condoms	1 pack	0	0.11	0	0	0	0	0	0.11	0	0	0.15	0
Female condoms	1 pack	0	0	0	0	0	0	0	0	0	0	0.27	0
Intrauterine contraceptive device	1 device	0	1.00	0	0	0	0	0	2.43	0	0	0.00	0
Implants: levonorgestrel	1 device	0	0.67	0	0	0	0	0	0.81	0	0	NA	0
Implants: etonogestrel	1 device	0	0.89	0.33	0	0	0	0	1.62	0	0	NA	0
Diaphragm	1 device	NA	NA	0	0	NA	NA	NA	NA	NA	NA	NA	NA
Oxytocin injection	1 vial	0	0.20	0.22	0	0.32	0	0	0.49	0.24	0	0.42	0
Misoprostol	1 vial	0	0.18	0.16	0	0.65	0	0	0.49	0.16	0	1.19	0
Methyldopa	90 tablets	0	1.00	1.00	0	7.31	5.85	0	1.46	3.65	0	3.80	0
Magnesium sulphate 500 mg/2 ml	18 vials	0	8.81	0.70	0	11.70	0	NA	NA	NA	0	NA	0
Magnesium sulphate 500 mg/10 ml	18 vials	0	17.76	5.41	0	5.85	0	0	16.12	14.59	0	NA	0
Calcium gluconate	1 ampoule	0	0.29	0.22	0	NA	0	0	2.92	14.59	0	NA	0
Ferrous salt	30 tablets	0	0.07	0.07	NA	1.22	NA	NA	0.02	0.01	0	0.45	0
Folic acid	30 tablets	0	0.13	0.07	0	0.97	0.02	0	0.49	0.24	0	1.59	0
Ferrous Salt: Folic Acid 60/400	30 tablets	0	0.67	0.07	0	0.97	0	0	0.49	0.16	0	0	0
Ferrous Salt: Folic Acid 150/500	30 tablets	0	NA	0.07	0	0.49	0	0	0.49	0.26	NA	2.26	NA
Metronidazole	30 tablets	0	0.07	0.13	0	0.97	0.88	0	0.49	0.49	0	0.45	0
Clotrimazole pessary	6 tablets	0	0.28	0.11	0.06	0.89	0.65	0	2.92	2.92	0	0.79	0
Clotrimazole cream	1 tube	0	0.13	0.1`	0.49	0.97	0.81	0	0.49	0.41	0	0.45	0
Gentamicin	10 amp	0	0.45	0.67	1.30	4.06	4.87	0	3.24	2.43	0	1.51	0
Procaine benzylpenicillin	10 vials	NA	NA	NA	2.27	7.31	6.50	0	6.48	3.24	0	3.62	NA
Benzyl penicillin	10 vials	0	1.11	1.11	NA	NA	NA	NA	NA	NA	0	3.62	0
Benzathine benzylpenicillin	1 vial	0	0.11	0.17	0.15	0.97	0.65	0	0.49	0.49	0	0.42	0
Amoxicillin 125 mg	15 tablets	0	1.67	0.17	0.10	0.44	0.24	0	0.24	0.24	0	0.45	0
Amoxicillin 250 mg	15 tablets	0	0.10	0.03	0	0.49	0.26	0	0.24	0.49	0	0.23	0
Dexamethasone	1 vial	0	0.11	0.11	0	0.97	0.49	0	0.41	0.32	0	0.60	0
Zinc syrup	1 bottle	0	0.33	0.22	NA	0.97	0.89	NA	0.41	NA	NA	0.68	NA
Zinc tablet	10 tablets	0	0.22	0.11	0	0.41	0.32	0	0.32	0.16	0	0.60	0
Zinc ORS co-pack	1 kit	0	0.22	0.13	0	0.32	0.02	0	0.32	0.24	0	0.06	0
ORS sachets 200 ml	1 sachet	0	0.03	0.	NA	0.16	NA	NA	NA	NA	0	0.27	NA
ORS sachets 500 ml	1 sachet	0	0.02	0.02	0	NA	NA	NA	NA	0.08	NA	NA	NA
ORS sachets 1 L	1 sachet	0	NA	NA	0	0.16	0.16	0	0.08	0.08	0	0.09	0

*PNFP* Private not-for-profit, *ORS* Oral rehydration salts

Uganda’s private sector had the most commodities that cost more than a day’s wage (*n* = 9), with a magnesium sulphate 500 mg/10 ml treatment costing more than 16 days’ wage. Two long-acting reversible contraceptives, levonorgestrel 750mcg and the intrauterine contraceptive device, also cost more than a day’s wage. Kenya and Tanzania had 4 and 6 commodities, respectively, that cost more than a day’s wage in the private sector, with a magnesium sulphate treatment also costing the most days. Zambia’s private sector had 7 commodities that cost more than a day’s wage; all were maternal health commodities or antibiotics. Affordability patterns in the PNFP sector, although slightly better, were comparative to their private sector counterparts in Kenya, Tanzania and Uganda, with many of the commodities that cost more than a day’s wage in the PNFP sector also costing more than a day’s wage in the private sector.

### Accessibility

**Fig. 2:**
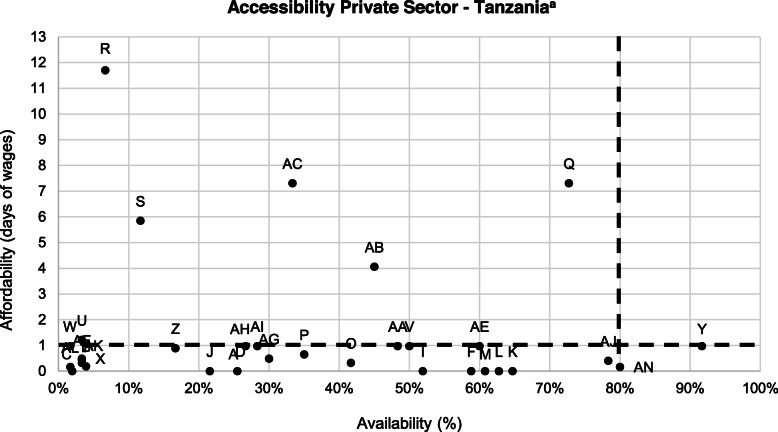
Accessibility of SRHC in Tanzania’s private sector.

Accessibility was low across the countries. In the public sector, where medicines are often provided free of charge, Kenya and Zambia had the highest accessibility, with six commodities considered accessible, followed by Tanzania (four commodities) and Uganda (two commodities). Accessibility was lower in the private sector. For instance, in Tanzania only two SRHC were accessible: ORS sachets 1 L and metronidazole (Fig. 2). Six commodities were both unaffordable and available in less than 80% of facilities: ferrous salt (1.22, 3%), gentamicin (4.06, 45%), magnesium sulphate 500 mg/10 ml (5.85, 11.7%), procaine benzylpenicillin (7.31, 33%), methyldopa (7.31, 73%) and magnesium sulphate 500 mg/2 ml (11.70, 6.7%). However, the problem for most SRHC seems to be availability, and not affordability, as many commodities are not available in 80% or more of facilities but do cost less than a day’s wage.

In Zambia’s private sector three SRHC met the accessibility threshold (amoxicillin 125 mg, male condoms and metronidazole), in Kenya two did (dexamethasone and male condoms), while in Uganda only one commodity (metronidazole) met the threshold. In Uganda and Zambia, six SRHC were also both unaffordable (more than a day’s wage) and had a low availability (less than 80%). In Kenya this was the case for four commodities. The PNFP sector had similar accessibility patterns as the private sector in the countries, with two or three commodities considered accessible across the countries’ PNFP sectors. Please refer to Additional file 2 for detailed information per country and sector.

## Discussion

### Findings and implications

This study researched the availability, affordability, stock-outs and accessibility of more than fifty sexual and reproductive health commodities considered essential by the WHO, in four Eastern and Southern African countries. The research showed that overall availability of these commodities remains low at less than 50% in all sectors, areas and countries, with highest mean availability found in Kenyan public facilities (46.6%). Stock-outs were a common occurrence across the countries; average number of stock-out days per month ranged from 3 days in Kenya’s private and PNFP sectors, to 12 days in Zambia’s public sector. In the public sectors of Kenya, Uganda and Zambia, as well as in Zambia’s PNFP sector, all SRHC were free for the patient. In the remaining sectors magnesium sulphate was the least affordable SRHC. Accessibility was low across the countries, with Kenya’s and Zambia’s public sectors having six SRHC that met the accessibility threshold, while the private sector of Uganda had only one SRHC meeting the threshold.

Similar trends highlighting in which aspects access to SRHC is lacking and where there is room for improvement were observed in the four countries. Availability of levonorgestrel 750mcg, the emergency contraceptive, was for example low across the countries, and this finding is reflected in the trends of use [49–52]. Comparable to other studies, magnesium sulphate, critical in managing pre-eclampsia and eclampsia, also had a low availability in all countries, with an especially low availability in Zambia [28, 53, 54]. Medical devices also had a suboptimal availability: tubal ligation and vasectomy kits had a very low availability across the countries, while availability of ultrasound scans was shown to be variable, with a higher availability in Kenya’s and Uganda’s public sector than in Tanzania and Zambia. An important note to make on the ultrasound scan is that according to the NEMLs, in Kenya it is available starting at county hospitals and in Uganda starting at Health Centres IV, while in Tanzania and Zambia it ought to be available at lower level facilities as well [40–43].

Low availability of many of the SRHC is exacerbated in these four countries by regular stock-outs, which often last for a significant part of the month. Further, even though affordability does not seem to aggravate access issues in the public sectors, it does constitute a problem in the private and PNFP sectors, where people turn to if SRHC are unavailable in the public sector [55, 56]. In these sectors, affordability might pose an even bigger issue than illustrated in this research due to the fact that a large proportion of the population does not earn the wage of an LPGW. For instance, in Zambia an LPGW earns the equivalent of about 4.50 USD, while in 2016 36.1% of the population was living below the poverty line of 1.90 USD [47, 57].

Other health system challenges beyond the price and availability of the commodities at the health facility, which were not measured in this research, also influence accessibility. These challenges include policy and regulatory issues, infrastructural issues, lack of knowledge amongst the population and healthcare workers, cultural beliefs, and lack of skilled healthcare workers [25, 58–67]. The physical availability of an ultrasound scan, for example, does not mean it is routinely used or functional; lack of healthcare workers trained in its use, lack of electricity or high user costs are also barriers [61]. Use and acceptability of male and female sterilization is also dependent on lack of knowledge and negative attitudes of clients and healthcare workers, religious beliefs, fear of surgery and side effects, lack of equipment, long travel distances, and long waiting times [62–66, 68].

Barriers to access are also created by policies and regulations. When a commodity is expected to be provided only at higher levels, as is the case for ultrasound scans, it increases the distance patients have to travel and reduces access [60, 66, 68]. Related, a slightly higher use of the emergency contraceptive in Kenya (1.7%) than in the other countries (0.2–0.5%), might be explained by the fact that only in Kenya is this contraceptive available without a prescription [49–52]. Another example is that major barriers to the availability of magnesium sulphate previously identified in Zambia included lack of policy implementation, lack of procurement by the Ministry of Health and stock-outs at the central distributor [59]. Efforts from governments thus ought to focus on improving availability, affordability, geographical accessibility and quality of offered SRH services on the one hand, and SRH client and community education on the other hand.

Key to improving access to SRHC is strengthening the health system, with a specific focus on the supply chain. Stock-outs are a serious issue across the countries, and governments ought to ensure that stock management systems are in place in health facilities; this research showed that especially in Tanzania and Zambia, there are still a number of facilities who do not have stock cards or an electronic stock management system in place. Further, better quantification of medicines is needed, as stock-outs are partly caused by the use of estimations for the needed medicines, and not on previous consumption data, and anticipated burden and need [55, 69]. Improved stock management at the central level is also critical, as poor stock management at this level results in commodities not delivered for extended periods, or commodities delivered that have not been ordered [26, 69]. In line with this, the government needs to ensure timely payment of commodity suppliers, as irregular or delayed payments can lead to a delayed or diminished supply until payment is received [55, 70].

A tool that can be used by governments to improve availability is Universal Health Care (UHC) packages. At the moment, UHC is a priority on the countries’ development agenda, and governments are adopting and implementing UHC and UHC packages [71–74]. A simple way to increase availability of essential SRHC could be to include the SRHC in these packages.

To tackle the negative attitudes and lack of knowledge on use of family planning services among the community, and to improve healthcare workers’ knowledge on SRHC and their professionalism, community sensitisation programmes and healthcare workers refresher trainings should be promoted and implemented. A review has shown that programmes using a combination of healthcare worker training, opening youth friendly health corners in health facilities, and sensitisation in communities and schools and through the media are most effective in improving knowledge of and demand for SRHC [75].

### Strengths and limitations

The major strength of this study is the use of a standardized and validated methodology which allows for the measurement of medicine prices, availability and affordability [30, 31]. This research also used a combined measure of availability and affordability to illustrate accessibility, as first introduced by Ewen et al. [76]. The added value of this combined measure is that it easily illustrates in what respects the WHO’s target for availability and affordability of essential medicines is falling short [44]. However, the used methodology also has some limitations, which have been previously identified [77]. One of the limitations of the methodology is collection of availability data at only one point in time. This research included the collection of commodity stock-out information with the aim to provide a more accurate picture of the availability situation across time. However, stock-data was collected only for the previous 6 months, so some seasonal or financial year differences might not have been captured.

The methodology further calculates affordability using the wage of an LPGW to allow for easy comparisons of data across countries [30]. However, in many developing countries, the wage of an LPGW is higher than what a large proportion of the population earns. It is therefore likely that the affordability projections here are an over-estimation of the actual affordability. Further, in this study ‘accessibility’ should be construed in the basic sense of the word as it is explained here, and it should be noted that socioeconomic factors as potential determinants for low access were not taken into account as data on this went beyond the scope of the research. When considering the recommendations, this should be kept in mind.

Another limitation of the existing methodology is that it only collects data for the outcome measures for one dosage form or strength, while a commodity might be available in other dosage forms. This research tried to mitigate this by aligning the surveyed commodities’ strengths and dosage forms to those on the countries’ corresponding NEMLs. When a commodity was listed with multiple dosage forms or strengths, they were all included. Further, in the PNFP sector, availability of family planning commodities is likely to be an overestimation of the actual situation in the countries. In this sector, only facilities offering family planning services were included in the analysis for contraceptives availability. Lastly, in this research, the oral contraceptive is a combined measure of multiple formulations and strengths. Availability seems high, but this is the availability of any oral contraceptive, while for women it might make a difference which oral contraceptive is available. Switching on a regular basis between different oral contraceptives due to unavailability of the preferred method can easily lead to side effects or discontinuation of use.

## Conclusion

This research has shown that accessibility of essential commodities for sexual and reproductive health remains a challenge in Eastern and Southern Africa. Low availability of SRHC in the public sector is compounded by regular stock-outs, which may force patients to seek care in private and PNFP sector facilities, where availability is also often low, where some services might not be offered or where the commodities might be unaffordable to a large proportion of the population. This research indicates that health system strengthening and community sensitisation is needed to ensure adequate access to essential SRHC. The findings of this research should be used by national governments and policy makers as a starting point to identify where the gaps and shortcomings in their health systems lie, and what commodities need priority attention.

## Supplementary information

**Additional file 1: Table S1.** Surveyed sexual and reproductive health commodities. The sexual and reproductive health commodities that were surveyed in this research in the four countries.

**Additional file 2: Figure S1.** Accessibility of SRHC in Kenya, Tanzania, Uganda and Zambia, per sector. Accessibility of the sexual and reproductive health commodities per sector in Kenya, Tanzania, Uganda and Zambia.

## Data Availability

The datasets generated and analysed during the current study are not publicly available due to institutional requirements but are available from the corresponding author on reasonable request.

## Abbreviations

HAI: Health Action International
LPGW: Lowest-Paid Government Worker
NEML: National Essential Medicine List
ORS: Oral Rehydration Salts
PNFP: Private Not-For-Profit
SRH: Sexual and Reproductive Health
SRHC: Sexual and Reproductive Health Commodities
STI: Sexually Transmitted Infections
UHC: Universal Health Care
WHO: World Health Organization
