# Supplementary material for: Access to sexual and reproductive health commodities in East and Southern Africa: a cross-country comparison of availability, affordability and stock-outs in Kenya, Tanzania, Uganda and Zambia
Source: BMC Public Health. 2020 Jul 3;20:1053. doi: 10.1186/s12889-020-09155-w (PMC7333276; doi:10.1186/s12889-020-09155-w)
Supplement: Supplementary file 1 — Additional file 1: Table S1. Surveyed sexual and reproductive health commodities. The sexual and reproductive health commodities that were surveyed in this research in the four countries. [file 12889_2020_9155_MOESM1_ESM.pdf]

Additional file 1

Table 1. Surveyed sexual and reproductive health commodities.

Services	Commodities
**Family Planning**	
	Ethinylestradiol + levonorgestrel 30mcg/150mcg
	Ethinylestradiol + norethisterone 50mcg/1.0mg[a,b,d]
	Ethinylestradiol + norethisterone 35mcg/1.0mg[b,c]
	Ethinylestradiol + norethisterone 30mcg/30mcg[a,c,d]
	Ethinylestradiol + desorgestrel 50mcg/250mcg[a,b,d]
	Ethinylestradiol + desorgestrel 30mcg/15mcg[a,c,d]
	Levonorgestrel 30mcg
	Levonorgestrel 750mcg
	Medroxyprogesterone acetate 150ml in 1ml
	Medroxyprogesterone acetate 104ml in 1ml[b,d]
	Estradiol cypionate + medroxyprogesterone acetate 5mg/25mg[b,c,d]
	Norethisterone enanthate 200mg/ml in 1ml[a]
	Male condoms: 3 per pack
	Female condoms: 1 per pack
	Intrauterine contraceptive device
	Implants: levonorgestrel
	Implants: etonogestrel
	Diaphragm
**Maternal Health**	
	Oxytocin injection 10IU, 1ml
	Misoprostol 200mcg
	Methyldopa 250mg
	Magnesium sulphate 500mg/ 1ml[a,b,d]
	Magnesium sulphate 500mg/ 2ml
	Magnesium sulphate 500mg/ 10ml
	Calcium gluconate 100mg/ml in 10ml ampoule
	Ferrous salt 200mg
	Folic acid 5mg
	Ferrous Salt: Folic Acid 60mg/400mcg
	Ferrous Salt: Folic Acid 200mg/500mcg[a,b,d]
	Ferrous Salt: Folic Acid 150mg/500mcg[c]
**Antibiotics and Antifungals**	
	Metronidazole 200mg
	Clotrimazole pessary 100mg[a,b,d]
	Clotrimazole pessary 500mg[c]
	Clotrimazole cream 1%, 15g tube
	Gentamicin 80mg/2ml[a,b,d]
	Gentamicin 40mg/1ml[c]
	Procaine benzylpenicillin powder for injection 3MU[a,b,c]
	Procaine benzylpenicillin powder for injection 4MU[a,d]
	Benzyl penicillin powder for injection 600mg[b,d]
	Benzyl penicillin injection 5MU[a,b,c]
	Benzathine benzylpenicillin 2.4MU in 10ml[a]
	Benzathine benzylpenicillin 900mg[b,c,d]
	Amoxicillin 125mg/5ml syrup[a,b,c]
	Amoxicillin 125mg[d]
	Amoxicillin 250mg

Newborn and Child Health
Dexamethasone 4mg/ml
Zinc syrup 10mg in 5ml syrup
Zinc tablet 20mg
Zinc ORS co-pack 10mg tablet/1L
ORS sachets 200ml
ORS sachets 500ml
ORS sachets 1L
Safe Delivery Kit
Chlorhexidine 4%
**SRH medical devices**
Vasectomy kit
Tubal ligation kit
Manual Vacuum Aspiration (MVA) kit
Speculum
Cervical dilator
Incubator
Monitor
Ultrasound scan
Ventilator
Foetal scope
Resuscitator (adult size)
Resuscitator (infant size)
Bag and mask (size 0)
Suction device
Training mannequin for infant resuscitation

[a] not surveyed in Kenya
[b] Not surveyed in Tanzania
[c] Not surveyed in Uganda
[d] Not surveyed in Zambia
