# Supplementary material for: Access to sexual and reproductive health commodities in East and Southern Africa: a cross-country comparison of availability, affordability and stock-outs in Kenya, Tanzania, Uganda and Zambia
Source: BMC Public Health. 2020 Jul 3;20:1053. doi: 10.1186/s12889-020-09155-w (PMC7333276; doi:10.1186/s12889-020-09155-w)
Supplement: Supplementary file 2 — Additional file 2: Figure S1. Accessibility of SRHC in Kenya, Tanzania, Uganda and Zambia, per sector. Accessibility of the sexual and reproductive health commodities per sector in Kenya, Tanzania, Uganda and Zambia. [file 12889_2020_9155_MOESM2_ESM.pdf]

Additional file 2

Figure 1. Accessibility of SRHC in Kenya, Tanzania, Uganda and Zambia, per sector.

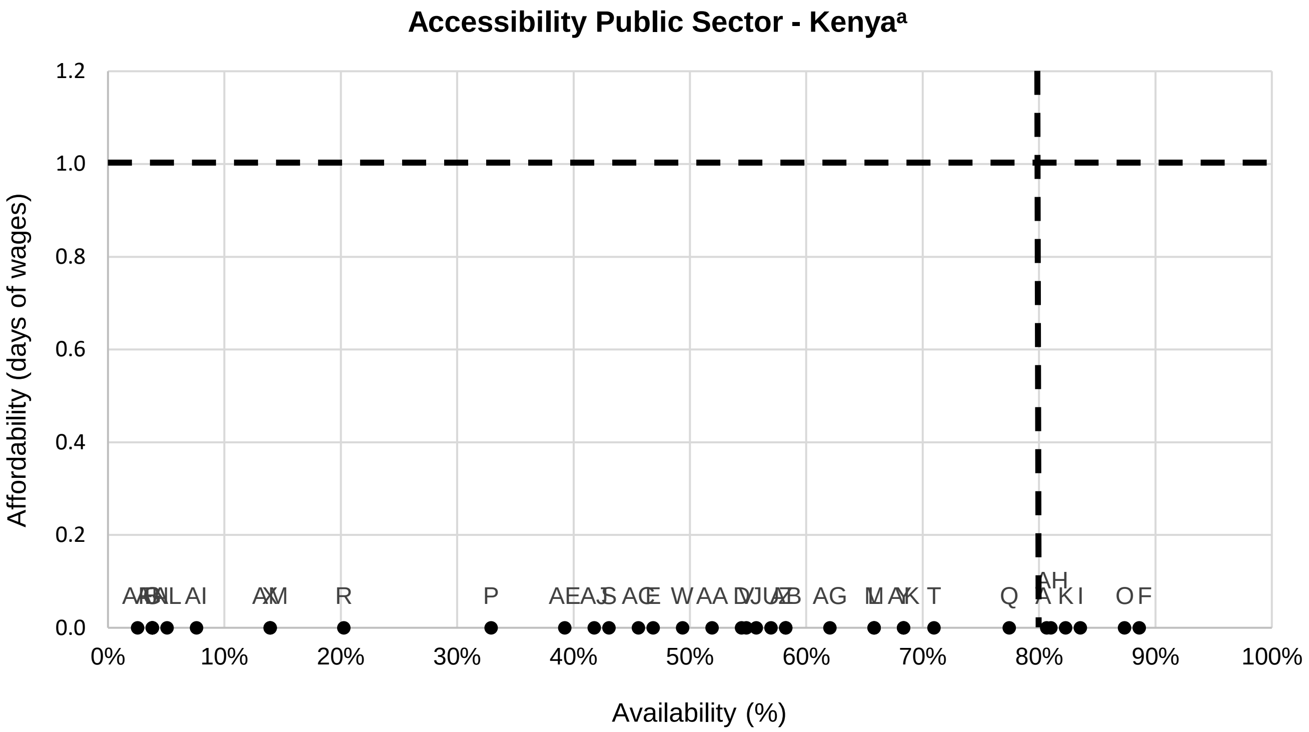

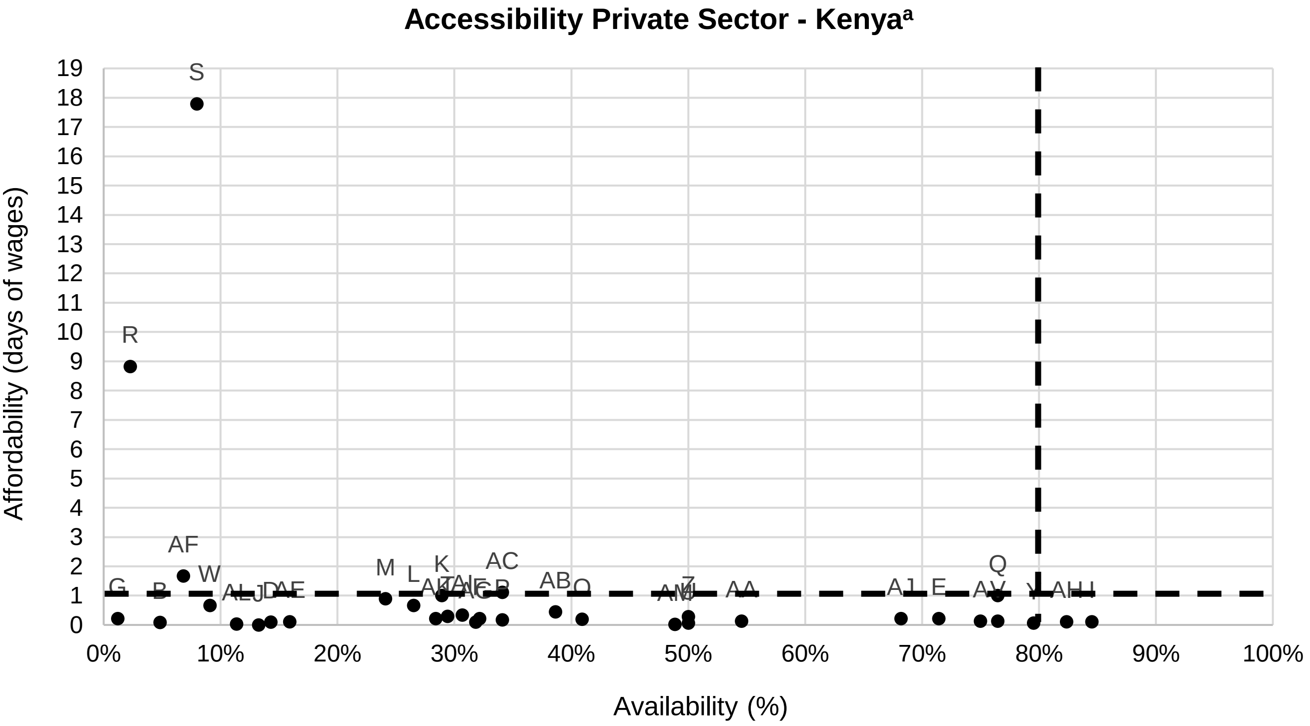

Accessibility Private Not-For-Profit Sector - Kenya[a]

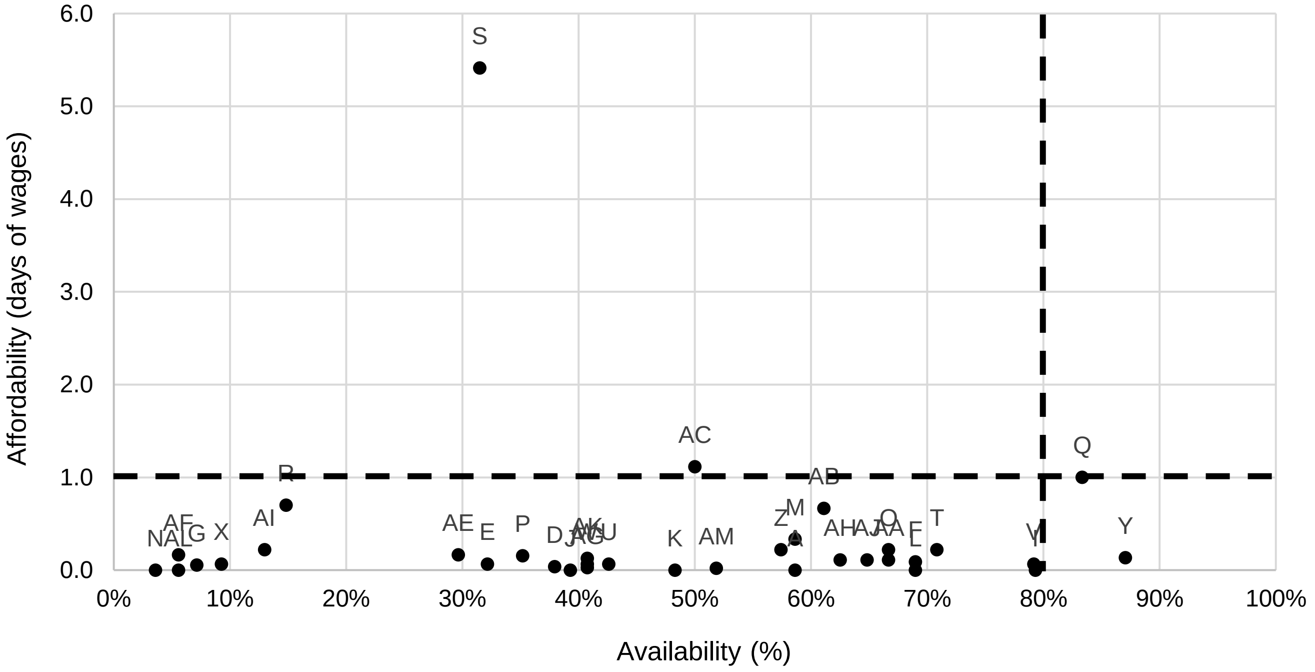

Accessibility Public Sector - Tanzania[a]

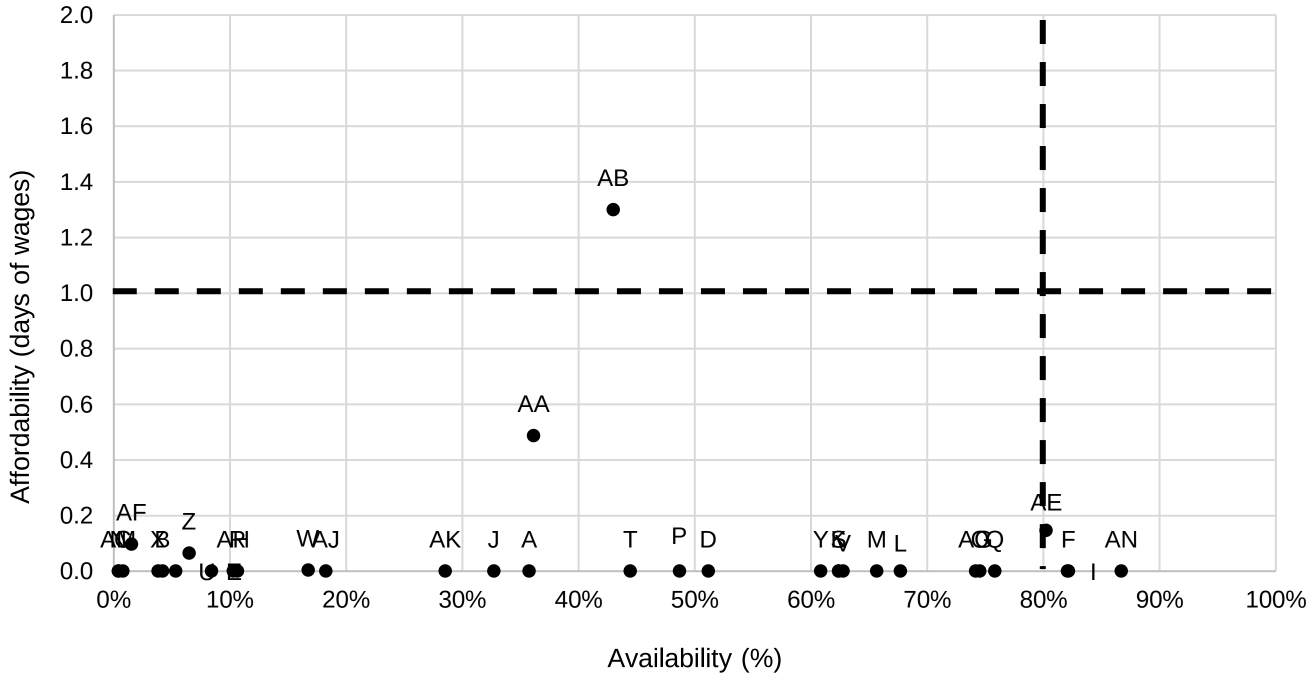

Accessibility Private Not-For-Profit Sector - Tanzania[a]

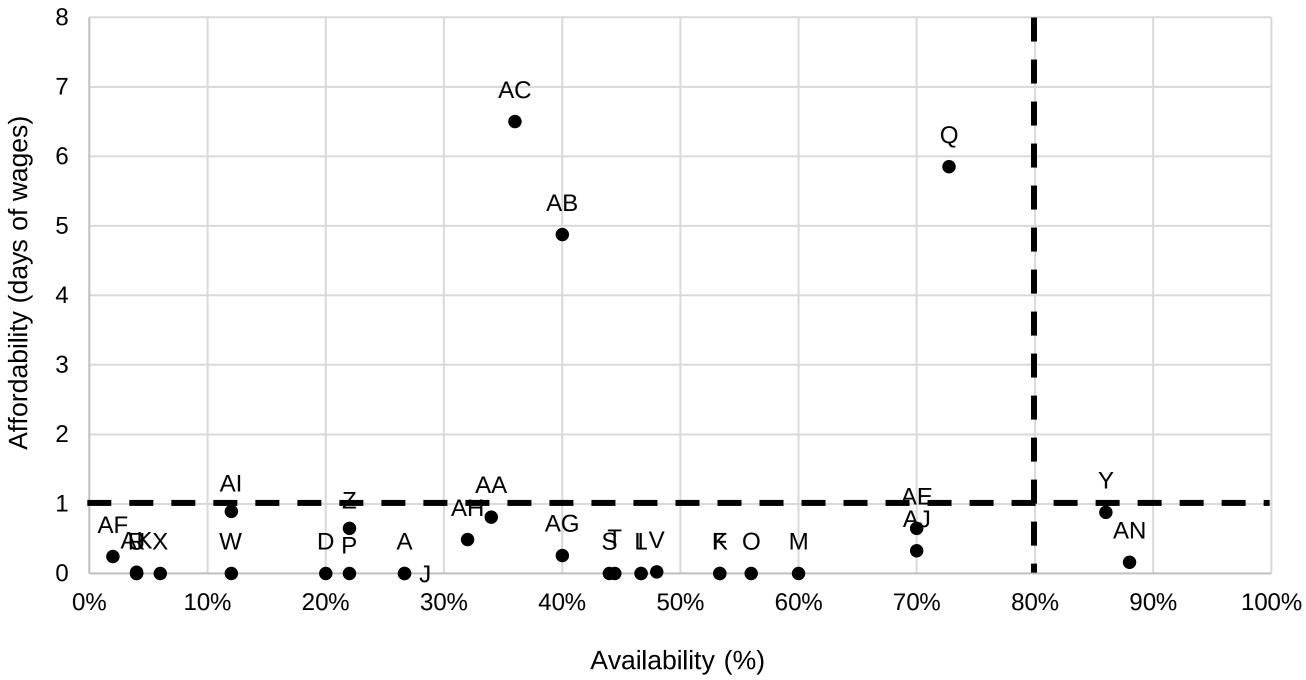

Accessibility Public Sector - Uganda[a]

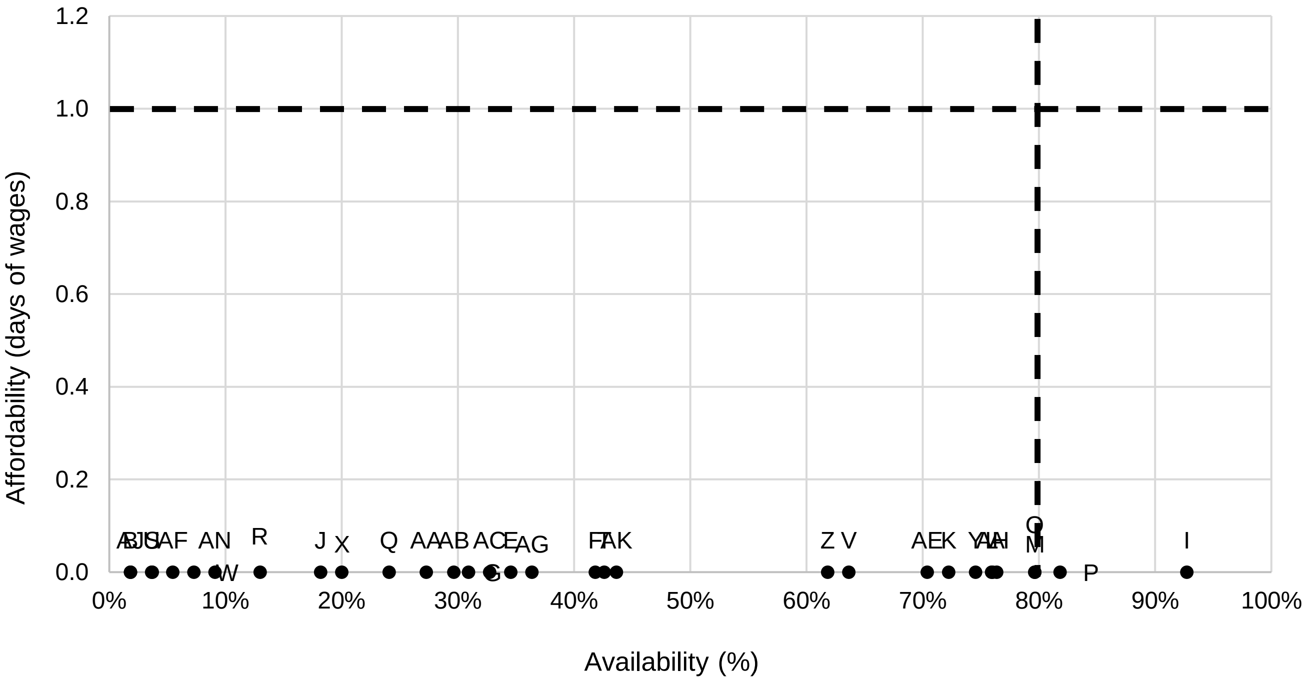

Accessibility Private Sector - Uganda[a]

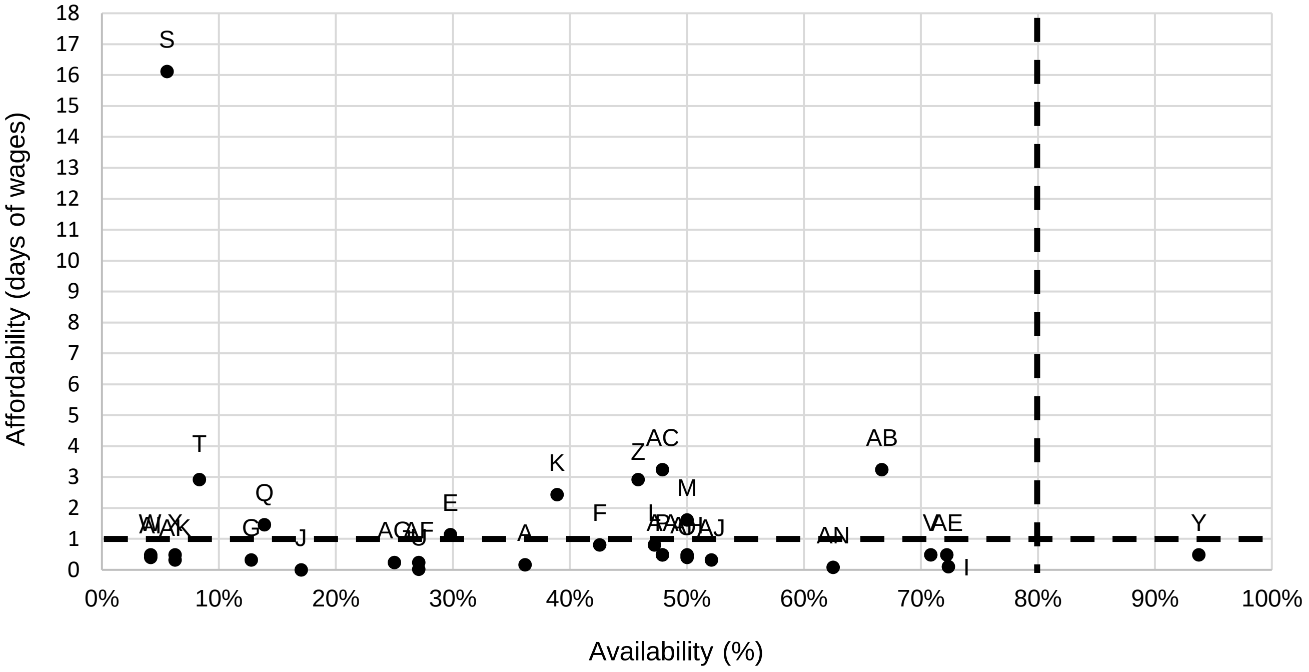

Accessibility Private Not-For-Profit Sector - Uganda[a]

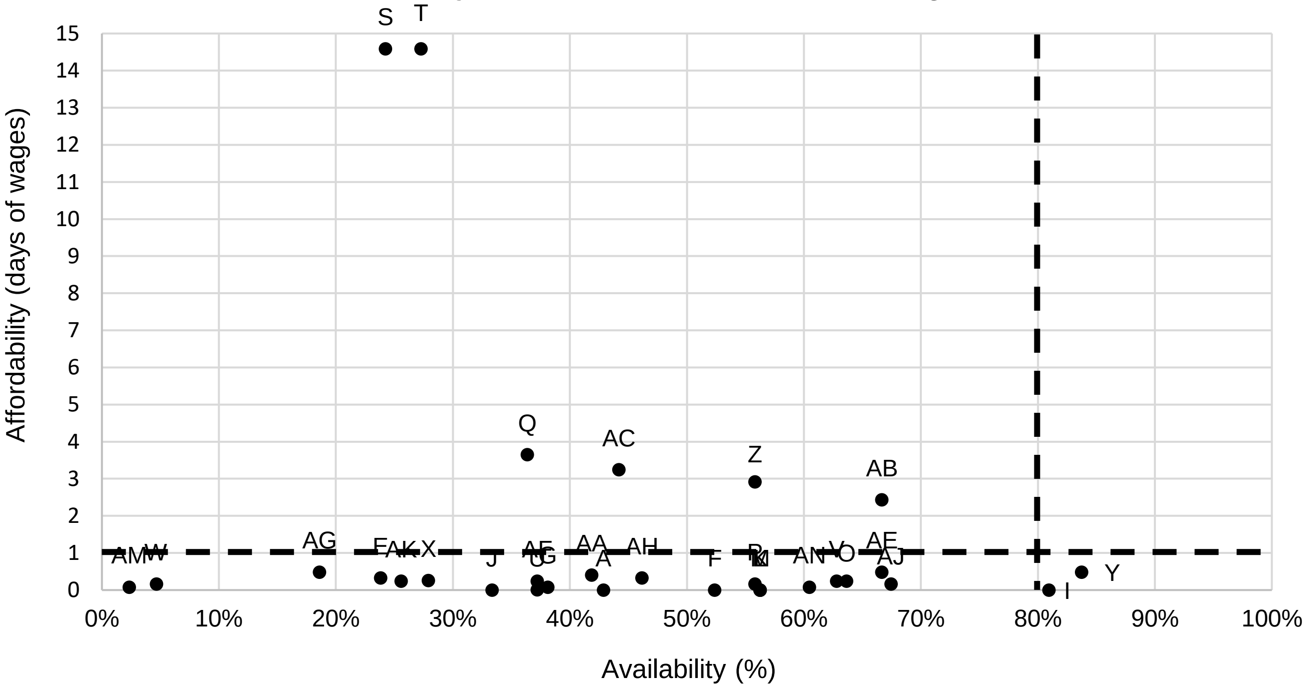

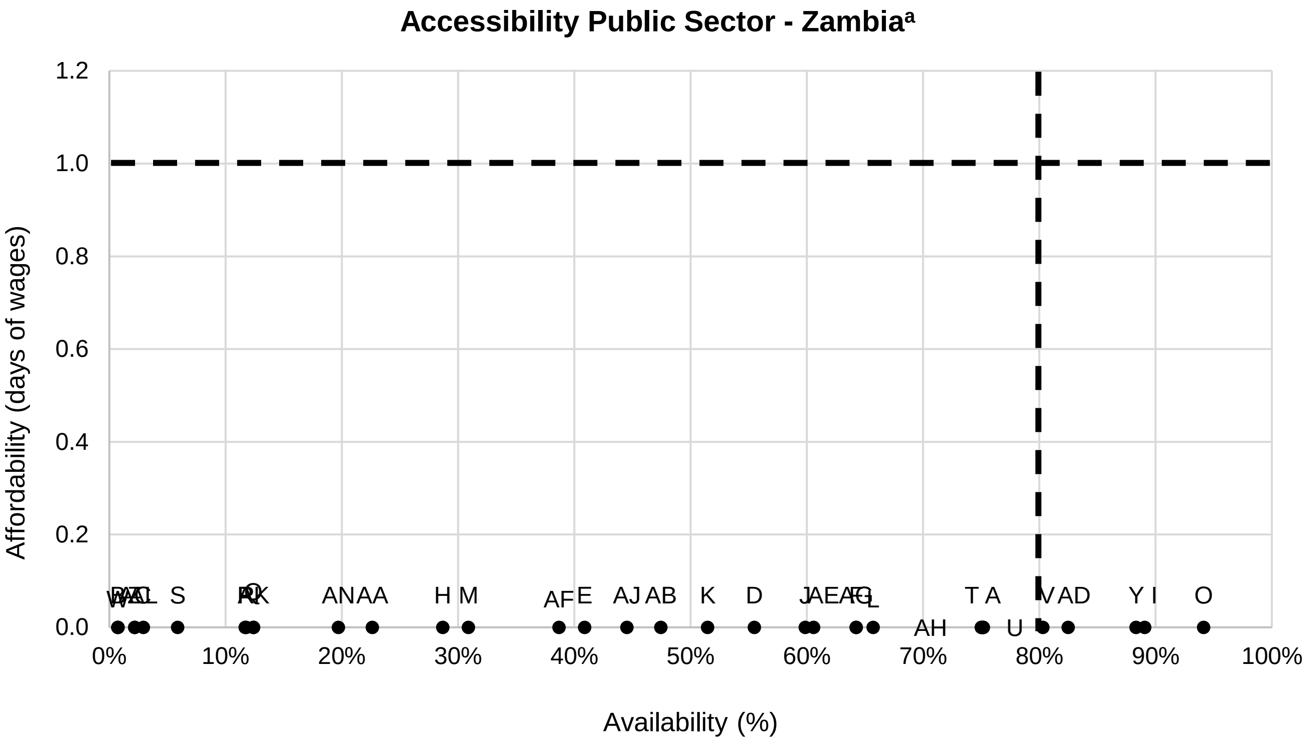
Accessibility Public Sector - Zambia[a]
Affordability (days of wages)
1.2
1.0
0.8
0.6
0.4
0.2
0.0
0%
10%
20%
30%
40%
50%
60%
70%
80%
90%
100%
Availability (%)
S
ANAA
H M
AF E
AJ AB
K
D
AH
T A
U
V AD
Y I
O

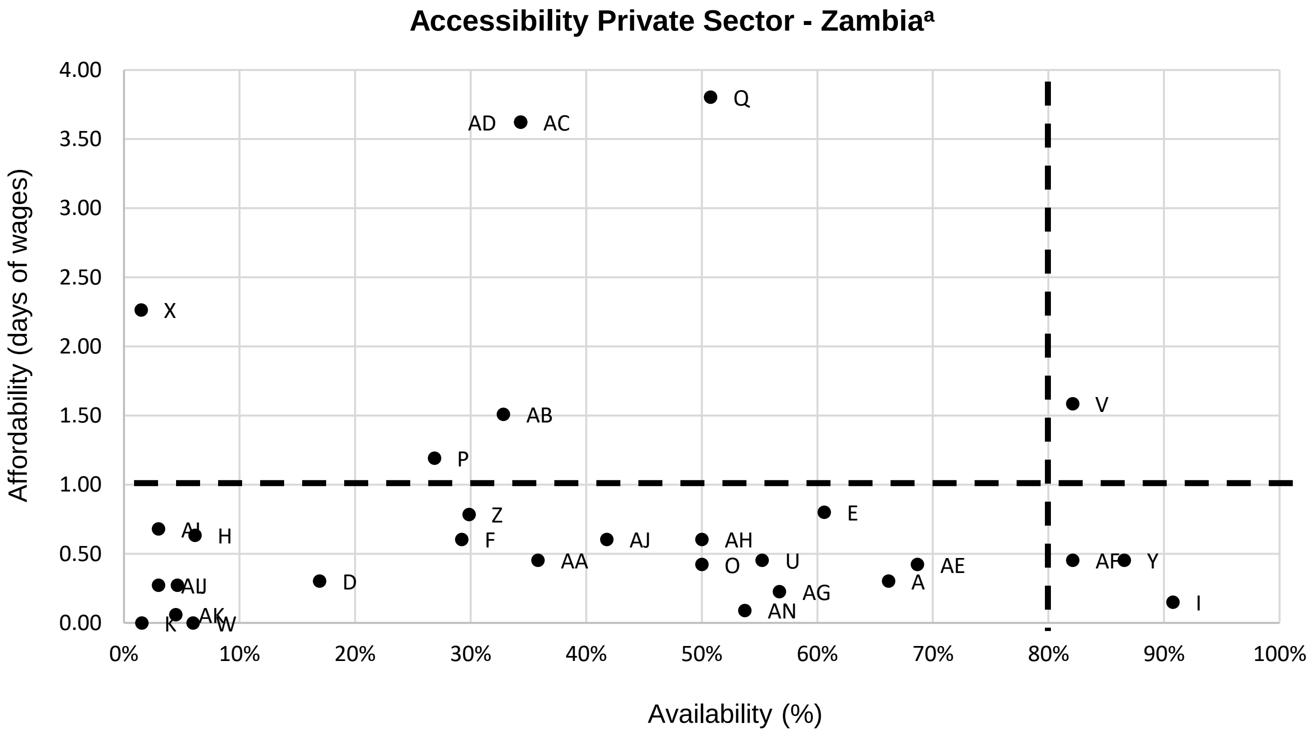
Accessibility Private Sector - Zambia[a]
Affordability (days of wages)
4.00
3.50
3.00
2.50
2.00
1.50
1.00
0.50
0.00
0%
10%
20%
30%
40%
50%
60%
70%
80%
90%
100%
Availability (%)
Q
AD
AC
X
AB
V
P
Z
E
H
F
AJ
AH
AA
O
U
AE
AF
Y
D
AG
A
I
AN
K
W

Accessibility Private Not-For-Profit Sector - Zambia[a]

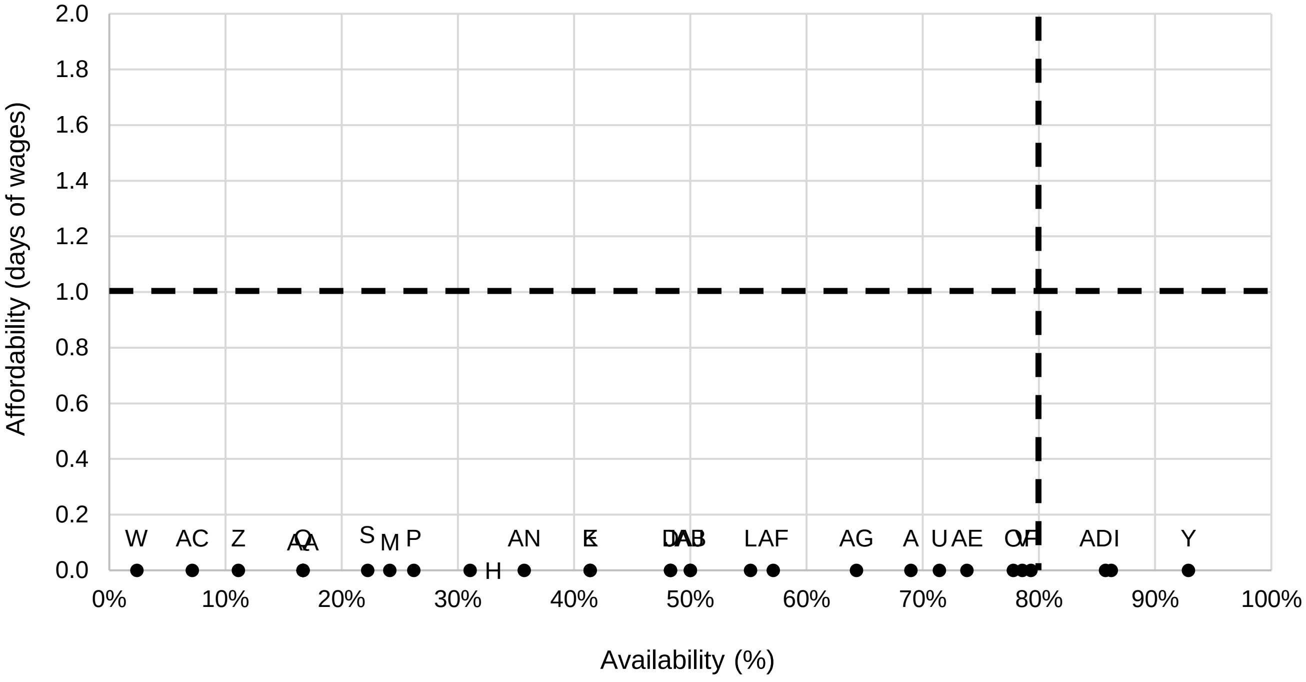

[a]A: Ethinylestradiol + levonorgestrel; B: Ethinylestradiol + norethisterone; C: Ethinylestradiol + desorgestrel; D: Levonorgestrel 30mcg; E: Levonorgestrel 750mcg; F: Medroxyprogesterone acetate 150ml; G: Medroxyprogesterone acetate 104ml; H: Norethisterone enanthate; I: Male condoms; J: Female condoms; K: Intrauterine contraceptive device; L: Implants: levonorgestrel; M: Implants: etonogestrel; N: Diaphragm; O: Oxytocin injection; P: Misoprostol; Q: Methyldopa; R: Magnesium sulphate 500mg/ 2ml; S: Magnesium sulphate 500mg/ 10ml; T: Calcium gluconate; U: Ferrous salt; V: Folic acid; W: Ferrous Salt: Folic Acid 60/400; X: Ferrous Salt: Folic Acid 150/500; Y: Metronidazole; Z: Clotrimazole pessary; AA: Clotrimazole cream; AB: Gentamicin; AC: Procaine benzylpenicillin; AD: Benzyl penicillin; AE: Benzathine benzylpenicillin; AF: Amoxicillin 125mg; AG: Amoxicillin 250mg; AH: Dexamethasone; AI: Zinc syrup; AJ: Zinc tablet; AK: Zinc ORS co-pack; AL: ORS sachets 200ml; AM: ORS sachets 500ml; AN: ORS sachets 1L.
